# The ability of non-physician health workers to identify chest indrawing to detect pneumonia in children below five years of age in low- and middle-income countries: A systematic review and meta-analysis

**DOI:** 10.7189/jogh.13.04016

**Published:** 2023-02-03

**Authors:** Ahad Mahmud Khan, Saima Sultana, Salahuddin Ahmed, Ting Shi, Eric D McCollum, Abdullah H Baqui, Steve Cunningham, Harry Campbell, RESPIRE Collaboration

**Affiliations:** 1 Usher Institute, University of Edinburgh, Edinburgh, UK; 2 Projahnmo Research Foundation, Dhaka, Bangladesh; 3 Eudowood Division of Paediatric Respiratory Sciences, Department of Paediatrics, School of Medicine, Johns Hopkins University, Baltimore, Maryland, USA; 4 Department of International Health, Johns Hopkins Bloomberg School of Public Health, Baltimore, Maryland, USA; 5 Centre for Inflammation Research, University of Edinburgh, Edinburgh, UK

## Abstract

**Background:**

Non-physician health workers play a vital role in diagnosing and treating pneumonia in children in low- and middle-income countries (LMICs). Chest indrawing is a key indicator for pneumonia diagnosis, signifying the severity of the disease. We conducted this systematic review to summarize the evidence on non-physician health workers' ability to identify chest indrawing to detect pneumonia in children below five years of age in LMICs.

**Methods:**

We comprehensively searched four electronic databases, including MEDLINE, Embase, Web of Science, and Scopus, and reference lists from the identified studies, from January 1, 1990, to January 20, 2022, with no language restrictions. Studies evaluating the performance of non-physician health workers in identifying chest indrawing compared to a reference standard were included. We used the Quality Assessment of Diagnostic Accuracy Studies (QUADAS-2) tool to assess the methodological quality of the selected studies and conducted a meta-analysis following a bivariate random effects model to estimate the pooled sensitivity and specificity.

**Results:**

We identified nine studies covering 4468 children that reported the accuracy of a non-physician health worker in identifying chest indrawing. Most studies were conducted in the 1990s, based at health facility settings, with children aged 2-59 months, and with pediatricians/physicians as the reference standard. Using the QUADAS-2, we evaluated most studies as having a low risk of bias and a low concern regarding applicability in all domains. The median sensitivity, specificity, positive predictive value, and negative predictive value were 44%, 97%, 55%, and 95%, respectively. We selected five studies for the meta-analysis. The pooled sensitivity was 46% (95% confidence interval (CI) = 37-56), and the pooled specificity was 95% (95% CI = 91-97).

**Conclusions:**

We found the ability of non-physician health workers in LMICs in identifying chest indrawing pneumonia is relatively poor. Appropriate measures, such as targeted identification and training, supportive supervision, regular performance assessment, and feedback for those who have a poor ability to recognize chest indrawing, should be taken to improve the diagnosis of pneumonia in children. New studies are needed to assess the new generation of health workers.

**Registration:**

PROSPERO (CRD42022306954).

Pneumonia is a leading cause of mortality worldwide in children under five years of age. In 2019, there were approximately 740 000 child deaths due to pneumonia globally [1]. There were an estimated 68 million pneumonia episodes in 2016, equivalent to 0.11 cases per child-year. A notable discrepancy has been observed in the incidence of pneumonia between high-income and low- and middle-income countries (LMICs) [2]. Pneumonia is a key reason for hospital admissions of children and is a substantial burden on health systems [3].

In LMICs, child pneumonia is usually poorly understood by caregivers, and care-seeking for treatment is not adequate [4]. Pneumonia is further underdiagnosed and undertreated due to the low doctor-to-population ratio [5]. Access to doctors and hospitals is difficult [6,7] and treatment costs are often not affordable [8]. Consequently, a large proportion of pneumonia cases are diagnosed and treated out of hospitals by non-physician health workers [9]. These health workers apply pragmatic case management algorithms to diagnose, treat, and refer children suspected to have pneumonia during household visits or in community-level health facilities [10,11]. The role of health workers in community-based pneumonia case management has had a significant impact on lowering child mortality [12].

The World Health Organisation (WHO) Integrated Management of Childhood Illness (IMCI) guidelines primarily use fast breathing and chest indrawing to diagnose pneumonia in children. The health worker observes the child’s chest to identify fast breathing and chest indrawing [13,14]. Evidence shows that health workers can identify fast breathing with moderate accuracy [15]. A child is identified to have chest indrawing if the tissue below the lower chest wall moves inward when the child inspires (**Figure 1**) [16]. This clinical sign can occur when the lungs are inflamed from an infectious process and have poor lung compliance [17]. Although chest indrawing is insufficient for diagnosing pneumonia, this signifies the severity of pneumonia and might be useful in detecting children at risk of hypoxaemia [18]. Identifying chest indrawing can be challenging for health workers [19]. The possible reason could be the low prevalence of chest indrawing cases in the population [20,21]. The characteristics of the training received by the health workers have an effect on their performance [22], often leading to misdiagnosis of pneumonia and incorrect treatment [19].

**Figure 1:**
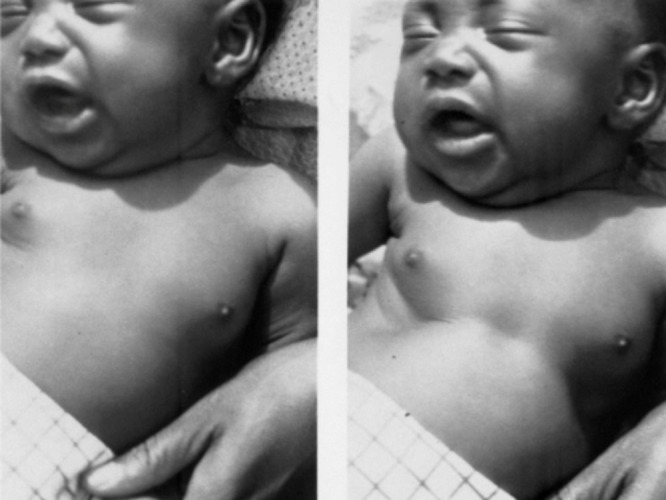
A child with chest indrawing. Reproduced with permission from World Health Organization.

The diagnosis and management of pneumonia in LMICs depends on health workers' capability to accurately identify chest indrawing. Regardless of existing literature assessing the performance of health workers in identifying chest indrawing, to the best of our knowledge, the evidence has never been systematically collated. Most of the existing literature involves studies with small samples. Therefore, a systematic review could provide more powerful evidence impacting clinical practice and health care policy. In this review, we summarized the evidence on how accurately non-physician health workers can identify chest indrawing in under-five children with suspected pneumonia in LMICs.

## METHODS

We conducted this systematic review and meta-analysis in accordance with the Preferred Reporting Items for Systematic Reviews and Meta-analyses (PRISMA) 2020 [23] and the Preferred Reporting Items for Systematic Reviews and Meta-analyses of Diagnostic Test Accuracy Studies (PRISMA-DTA) guidelines [24] following the guidance provided in the Handbook for Diagnostic Test Accuracy (DTA) Reviews of Cochrane [25]. We established the review methods prior to conducting it and prospectively registered it with PROSPERO (registration number CRD42022306954; January 26, 2022) [26].

### Population, index test, and reference standard

The target participants were children younger than five years assessed for chest indrawing in the community or a health facility. The index test was chest indrawing assessment by a non-physician health worker (e.g. community health worker, medical assistant, nurse, nursing assistant). The reference standard was assessment by a human expert, defined as a pediatrician, general physician, or an IMCI expert assessor.

### Search strategy

We developed a search strategy comprising medical subject headings (MeSH) terms and keywords. We systematically searched the following electronic databases for relevant studies published from January 1, 1990, until January 20, 2022: MEDLINE (via Ovid), Embase (via Ovid), Web of Science, and Scopus. We did not search for older studies considering that the IMCI strategy was launched in the 1990s. The detailed search strategy for each database is available in Table S1 in the **Online Supplementary Document**. We searched the identified studies’ reference lists to avoid missing relevant studies. The search strategy did not include any filters or limitations and we included studies published in any language. An expert librarian reviewed the search strategy.

### Study eligibility

We focused on studies that evaluated the performance of health workers in identifying chest indrawing against a reference standard. We included studies when all the following criteria were met:

1. Identification of chest indrawing by non-physician health workers.
2. Evaluation of the accuracy of identifying chest indrawing by a reference standard.
3. The age of the participants was below five years.
4. Carried out in LMICs [27].

The exclusion criteria were as follows:

1. Non-human subjects or mechanically ventilated subjects.
2. Lack of information on the reference standard.
3. Videotaped subjects were assessed by health workers.
4. Not possible to disaggregate data of chest indrawing.
5. Not possible to disaggregate data of under-five children.

### Study selection and data extraction

We imported the search results from different databases into Covidence Systematic Review software [28] and removed duplicates, after which two reviewers (AMK and SS) independently screened the retrieved studies’ titles and abstracts. Both reviewers independently read full papers of potentially relevant articles according to the eligibility criteria. The same reviewers extracted data independently using a structured form (Table S2 in the **Online Supplementary Document**), which included the following information: author, year, study location, study setting, sampling method, number of participants, index test, and reference standard. We also extracted data on true positive (TP), false positive (FP), false negative (FN), true negative (TN), sensitivity, specificity, positive predictive value (PPV), and negative predictive value (NPV) or calculated them from reported data. If relevant data were missing or not reported, we contacted the corresponding author by e-mail. We entered the data into a Microsoft Excel spreadsheet. We resolved disagreements for both the literature screening and the data extraction through discussion until we reached a consensus.

### Quality assessment

We used the Quality Assessment of Diagnostic Accuracy Studies (QUADAS-2) tool (Table S3 in the **Online Supplementary Document**) to assess the methodological quality of all studies. The tool includes four risk of bias domains and three domains of applicability [29]. Each domain has an overall judgment of “low risk of bias” if it was judged as “low” in all signaling questions. In contrast, the domain would be judged as “high risk of bias” if it was judged “high” in one or more signaling questions. We used the “unclear” category if inadequate data were reported. Again, any discrepancy between reviewers was discussed until a consensus was reached. We used Review Manager (version 5.4) to generate the figure presented in this report.

### Data synthesis and analysis

We presented the sensitivity, specificity, PPV, NPV, and accuracy of each study and computed median values and interquartile ranges (IQRs). We conducted a meta-analysis following a bivariate random effects model with studies where TP, FP, FN, and TN data could be retrieved and where an adequate number of chest indrawing cases was present in the sample. We presented study diagnostic sensitivity and specificity estimates with 95% confidence intervals (CIs) in paired forest plots using a user-written command (midas) [30]. The heterogeneity between studies was assessed from coupled forest plots and using the *I^2^* statistics [31]. We used Stata (version 17.0) to perform the analyses.

## RESULTS

### Search results

**Figure 2:**
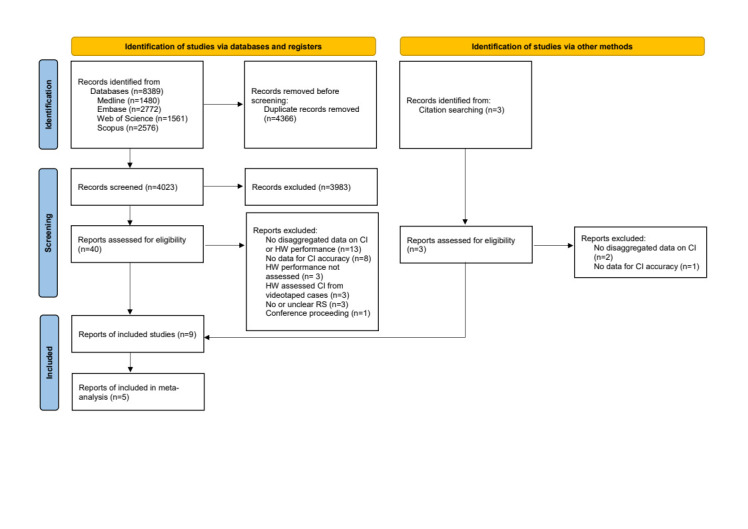
PRISMA flow diagram; CI – chest indrawing, HW – health worker, RS – reference standard.

A PRISMA flowchart summarizing the study selection process is presented in **Figure 2** [23]. The initial search retrieved 8389 records from all databases, forty of which we reviewed in full-text. We identified three additional articles from their reference lists for full-text review. We contacted the corresponding authors of 12 studies by email for relevant data not reported in the paper. Only three responded, but no one could provide any data. We included a total of nine studies in this review and five in the meta-analysis. The list of excluded studies and the reasons for exclusion are available in Table S4 in the **Online Supplementary Document**.

### Characteristics of the included studies

The major characteristics of the included studies are presented in **Table 1**. Out of nine studies, six were done before the year 2000 [21,32,33,36-38] and three studies were done after [20,34,35]. Most studies were conducted in Africa [21,32,34-38], two in Asia [20,36], and one in Oceania [33]. Only one study was conducted in a community setting [20] and the rest were conducted in health facility settings [21,32-38]. Two studies assessed young infants [20,32], while six assessed children aged 2-59 months [21,33-38]. The number of children per study ranged from 34 to 1405. The number of health workers ranged from 6 to 114. The workers were trained on the identification of pneumonia according to the WHO guidelines for a short duration at the beginning of the study. In seven studies, a pediatrician or a physician was the reference standard [20,21,32,34,36-38]. There was a short delay in assessment between the health worker and expert (i.e. expert assessment immediately after health worker assessment) in seven studies [21,32-34,36-38], while there was a long delay (i.e. reference standard assessed a few hours after health worker assessment) between assessments in two studies [20,35].

**Table 1:** Characteristics of the included studies

Author, year	Country	Setting	Population		Index test				Reference standard	
			**Age in months**	**Number of children**	**Performed by**	**Number of health workers**	**Qualification of health workers**	**Training**	**Performed by**	**Timing**
Baqui, 2009 [20]	Bangladesh	Community	0-1	288	CHW	41	Minimum 10th grade	6 wks	Physician	Long delay
Brady, 1993 [32]	Kenya	Health facility	0-2	200	Nursing students and school graduates	6	High school graduates, nursing students	1 wks	Paediatrician	Short delay
Brewster, 1993 [33]	Papua New Guinea	Health facility	1-59	223	Nurse and CHW	104	Not reported	Not reported	Evaluator	Short delay
Kelly, 2001 [34]	Kenya	Health facility	2-59	200, 216, and 414	CHW	100, 108 and 114	Not reported	3 wks	Study clinician	Short delay
Mulaudzi, 2015 [35]	South Africa	Health facility	2-59	34	Clinic health care worker	Not reported	Not reported	Not reported	Researcher	Long delay
Mulholland, 1992 [36]	Philippines and Swaziland	Health facility	2-59	308 and 291	Nursing assistant	Not reported	Not reported	1 d	Paediatrician	Short delay
Perkins, 1997 [21]	Kenya	Health facility	2-59	1405	Health worker	Not reported	High school graduates	Not reported	Physician	Short delay
Simoes, 1992 [37]	Swaziland	Health facility	2-59	331 and 304	Nursing assistant & nurse	3 and 6	Not reported	Not reported	Paediatrician	Short delay
Simoes, 1997 [38]	Ethiopia	Health facility	2-59	254	Nurse	6	Not reported	9 d	Paediatrician	Short delay

CHW – community health worker, wks – weeks, d – days

### Methodological quality of included studies

**Figure 3:**
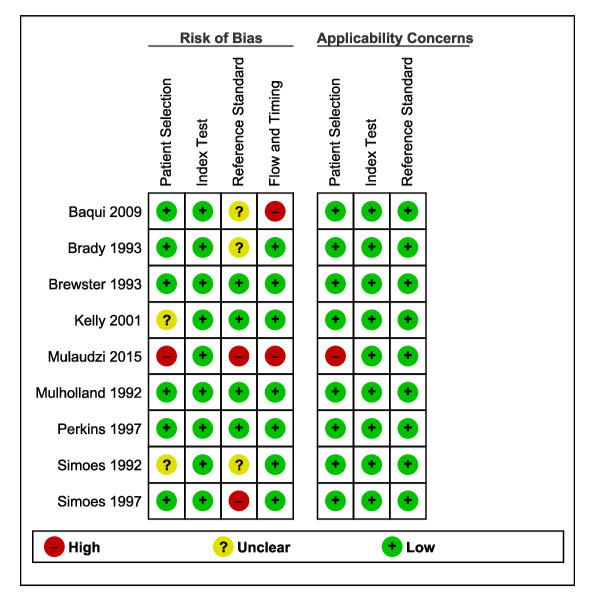
Risk of bias and applicability concerns summary: review authors’ judgements about each domain for each included study.

**Figure 3** presents the risk of bias and concerns regarding the applicability of the selected studies. For patient selection, we rated one study as having high risk of bias because of convenient sample selection [35]. Two studies had unclear information on the sampling method, so we judged them as having unclear risk of bias [34,37]. For the index test, we rated all studies as low risk of bias, as the health workers were blinded to the finding of the reference standard [20,21,32-38]. For the reference standard, we rated two studies as high risk of bias as the reference standard was not blinded [35,38] and three studies as unclear risk of bias due to unclear reporting on blinding [20,32,37]. For patient flow and timing, we evaluated two studies as having a high risk of bias because of a prolonged delay between the index test and reference standard [20,35]. Overall, the studies had low concerns regarding applicability for all domains [20,21,32-38]. The concern in one study was related to inclusion criteria for patient selection [35].

### Accuracy in chest indrawing identification by health workers compared to reference standard

The summary results of all included studies are presented in **Table 2****.** The median sensitivity, specificity, PPV, NPV, and accuracy are reported in **Table 3**. The median sensitivity and specificity were 44% and 97%, respectively.

**Table 2:** Studies reporting health worker identification of chest indrawing compared to reference standards

Author, year	Included participants		Age (months)	Sample prevalence	Sensitivity (95% CI)	Specificity (95% CI)	Positive predictive value (95% CI)	Negative predictive value (95% CI)	Accuracy (95% CI)
Baqui, 2009 [20]	All neonates visited in the households		0-1	1/287 = 0.003	0/1 = 0.00	287/287 = 1.00 (0.99-1.00)	0/0	287/288 = 0.99 (0.99-1.00)	287/288 = 0.99 (0.98-1.00)
Brady, 1993 [32]	Children with cough, fever or ‘not feeling well’ brought to hospital		0-2	39/200 = 0.20	15/39 = 0.38 (0.23-0.55)	144/161 = 0.89 (0.84-0.94)	15/32 = 0.47 (0.33-0.62)	144/168 = 0.86 (0.82-0.89)	159/200 = 0.80 (0.73-0.85)
Brewster, 1993 [33]	Children with cough or shortness of breath brought to the facility		1-59	33/223 = 0.15	11/33 = 0.33 (0.18-0.52)	175/190 = 0.92 (0.87-0.96)	11/26 = 0.42 (0.27-0.59)	175/197 = 0.89 (0.86-0.91)	186/223 = 0.83 (0.78-0.88)
Kelly, 2001 [34]	Children with any acute illness presented at hospital	First evaluation	2-59	-	30/50 = 0.60 (0.46-0.74)	-	-	-	-
		Second evaluation			7/37 = 0.19 (0.06-0.32)	-	-	-	-
		Third evaluation			0.53 (0.28-0.78)	-	-	-	-
Mulaudzi, 2015 [35]	Children with cough or difficult breathing referred from primary health center to hospital		2-59	-	-	-	2/11 = 0.18	-	-
Mulholland, 1992 [36]	Children with cough or breathing difficulty brought to the hospital	Philippines	2-59	28/308 = 0.09	13/28 = 0.46 (0.28-0.66)	241/280 = 0.86 (0.82-0.90)	13/52 = 0.25 (0.17-0.35)	241/256 = 0.94 (0.92-0.96)	254/308 = 0.83 (0.78-0.87)
		Swaziland		11/291 = 0.07	8/19 = 0.42 (0.20-0.67)	267/272 = 0.98 (0.96-0.99)	8/13 = 0.62 (0.37-0.82)	267/272 = 0.96 (0.94-0.97)	275/291 = 0.95 (0.91-0.97)
Perkins, 1997 [21]	Children with cough brought to the hospital		2-59	160/1405 = 0.11	91/160 = 0.57 (0.49-0.65)	1204/1245 = 0.97 (0.96-0.98)	91/132 = 0.69 (0.62-0.76)	1204/1273 = 0.95 (0.94-0.95)	1295/1405 = 0.92 (0.91-0.94)
Simoes, 1992 [37]	Children with cough or difficult breathing presenting at hospital	Nursing assistant	2-59	41/332 = 0.12	14/41 = 0.34 (0.20-0.51)	284/290 = 0.98 (0.96-0.99)	14/20 = 0.70 (0.49-0.85)	284/311 = 0.91 (0.89-0.93)	298/331 = 0.90 (0.86-0.93)
		Nurse		38/304 = 0.13	26/38 = 0.68 (0.51-0.83)	254/266 = 0.96 (0.92-0.98)	26/38 = 0.68 (0.55-0.80)	254/266 = 0.96 (0.93-0.97)	280/304 = 0.92 (0.89-0.95)
Simoes, 1997 [38]	Children with cough or difficult breathing presenting at primary health center		2-59	-	0.62	0.98	-	-	-

CI – confidence interval

**Table 3:** Health worker identification of chest indrawing compared to reference standards

Statistics	Number of studies	Median	IQR
Sensitivity	8	0.44	0.33-0.61
Specificity	7	0.97	0.91-0.98
Positive predictive value	6	0.55	0.29-0.69
Negative predictive value	6	0.95	0.90-0.96
Accuracy	6	0.91	0.83-0.94

IQR – interquartile range

### Results of meta-analysis

**Figure 4:**
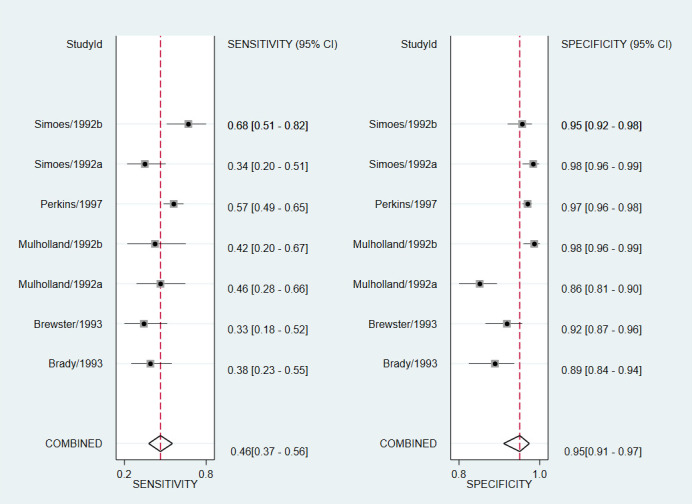
Accuracy of health workers’ chest indrawing identification compared to reference standards. Forest plots of individual and summary estimates of sensitivity and specificity.

Individual and summary estimates of sensitivity and specificity for the studies included in the meta-analysis are shown in **Figure 4**. The pooled sensitivity was 46% (95% CI = 37-56), the pooled specificity was 95% (95% CI = 91-97) and there was considerable heterogeneity (*I^2^* = 80%).

## DISCUSSION

Recognizing chest indrawing is a necessary skill for health workers in LMICs to diagnose and classify childhood pneumonia [13,14]. This systematic review demonstrated the performance of the non-physician health workers in identifying chest indrawing varied across the studies. The median sensitivity was 44%, with an IQR of 33%-61%; the pooled estimate of sensitivity was 46%. This low sensitivity implies that the health workers failed to identify chest indrawing among a substantial proportion of children who actually had chest indrawing. These children might have been diagnosed with pneumonia if they had other signs like fast breathing. Failure to identify chest indrawing may lead to underdiagnosis and inappropriate treatment. Sometimes the health workers were good at identifying chest indrawing. For example, in one study, the health workers identified chest indrawing with a sensitivity of 68% [37], and another study reported a sensitivity of 57% [21]. The median specificity and IQR were 97% and 91%-98%, respectively, with a meta-estimate of 95%. This high specificity indicates that most children who did not have chest indrawing were correctly identified. However, the high specificity does not necessarily mean that health workers' ability was excellent in excluding non-chest indrawing accurately. The possible reason might be the low prevalence of chest indrawing cases in the study population [39,40].

In studies conducted among children aged 2-59 months, the sensitivities ranged from 33% to 68%, and the specificity ranged from 86% to 98%. Brady et al. [32] conducted a study with infants aged 0-2 months, reporting a 38% sensitivity and a 89% specificity. We identified another study with neonates that fulfilled our eligibility criteria for this review. However, this study was not selected for the meta-analysis because of the insufficient number of chest indrawing cases [20]. Accurate identification of chest indrawing in young infants is often challenging for health workers. Mild chest indrawing is considered normal, often occurring in healthy infants, as the chest wall is not yet ossified and is more compliant [17]. However, severe chest indrawing is usually thought to be a very deep inward movement of the subcostal tissue and should be easier to identify. This is considered a danger sign for young infants [41]. Further studies are needed to evaluate the ability of health workers to identify this sign in young infants.

In LMICs, pneumonia signs are usually poorly recognized by parents and care-seeking at the facilities is low [4]. Community-based health workers play a key role in identifying pneumonia during household visits. Out of the nine included studies, only one was based in the community and had a single case of chest indrawing [20]. Therefore, the performance of health workers in identifying this sign at the community level could not be evaluated, necessitating large community-based studies.

In all of our included studies, the health workers’ ability to identify pneumonia signs was assessed using actual sick children, which should be ideal. However, children with pneumonia signs are often not readily available, and sick children may need to be treated immediately to safeguard their well-being. Therefore, some studies used videotaped subjects that may not have adequately depicted the signs [42-44]. The findings of those studies might have been different from those with actual children, and we excluded those studies from our review.

A human expert assessing chest indrawing in the same setting is usually considered the reference standard for health worker performance assessment. Seven of the nine included studies used a pediatrician or physician as a reference standard [20,21,32,34,36-38], and the other two did not report the expert’s qualification [33,35]. An expert is thought to be more accurate, but can over-assess or under-assess pneumonia signs. Hence, using expert assessment as a reference standard itself raises questions due to doubtful precision. Child assessment could be videotaped, and this video could be interpreted systematically by a panel of experts [45] or by a video-based automated system [46]. This could be an ideal reference standard for evaluating health workers in future studies and further research is needed in this area.

This review has several limitations. First, most of the selected studies were conducted in Africa, while two were in Asia and one in Oceania. This may limit the generalization of our findings to other LMICs. Second, most studies were conducted in the 1990s. We cannot determine anything about the current generation of health workers from our findings. We had identified some recent studies but could not include them in this review, as disaggregated chest indrawing data was irretrievable, even after we contacted the corresponding authors. Third, the health workers were trained before their assessment, which could affect the review results [47]. Their performance may change over time from training. The performance in the study might be better due to the direct observation by the evaluator [48] and might not reflect the health workers’ day-to-day performance. Lastly, health workers often assessed chest indrawing as a part of a larger study. Those studies may not have provided enough information on chest indrawing for being selected in this review or included in the meta-analysis.

We provide evidence on the necessity of improving the performance of health workers in identifying chest indrawing pneumonia. The health system constraints in LMICs include a lack of mentoring, supervision, and continuing development program for health workers. Additionally, there are limited auditing and quality improvement processes to evaluate the program [49]. The health workers’ performance could be improved by training combined with job aides, supportive supervision, regular performance evaluation, and feedback for those who have a poor ability to recognize chest indrawing [50]. A well-functioning monitoring process can identify health system constraints and can improve their performance [49]. The development of a video-based automated method for chest indrawing assessment [46] for health workers might be useful for identifying pneumonia cases in LMICs. An appropriate non-biased reference standard should be applied to assess health workers’ performance in identifying chest indrawing pneumonia.

## CONCLUSIONS

Through this review, we found that the performance of non-physician health workers in LMICs was relatively poor in identifying chest indrawing pneumonia. They could identify chest indrawing with poor sensitivity and reasonable specificity, showing a need for improvement. However, all the studies were conducted quite some time ago. New studies should be conducted to assess a new generation of health workers and to investigate possible reasons behind the challenges in identifying chest indrawing encountered by health workers. Appropriate measures should be taken to improve their performance for accurate diagnosis of pneumonia and appropriate treatment.

## Additional material


Online Supplementary Document
