# Supplementary material for: The ability of non-physician health workers to identify chest indrawing to detect pneumonia in children below five years of age in low- and middle-income countries: A systematic review and meta-analysis
Source: J Glob Health. 2023 Feb 3;13:04016. doi: 10.7189/jogh.13.04016 (PMC9894506; doi:10.7189/jogh.13.04016)
Supplement: Online Supplementary Document [file jogh-13-04016-s001.pdf]

ONLINE SUPPLEMENTARY DOCUMENT

Title: The ability of non-physician health workers to identify chest indrawing to detect pneumonia in children below five years of age in low- and middle-income countries: a systematic review and meta-analysis

Authors: Ahad Mahmud Khan, Saima Sultana, Salahuddin Ahmed, Ting Shi, Eric D McCollum, Abdullah H Baqui, Steve Cunningham, Harry Campbell; RESPIRE Collaboration

Table S1. Search strategies

**A. Name of Database: Ovid MEDLINE(R) 1946 to January Week 2 2022**		
1	exp Pneumonia/	228591
2	Respiratory Tract Infections/	40947
3	pneumon*.mp.	287799
4	(respiratory tract infection* or acute respiratory infection* or acute respiratory tract infection* or arti or lower respiratory infection* or lrti).mp.	56288
5	Infant, Newborn, Diseases/	38305
6	(child* illness* or newborn illness* or neonatal illness* or newborn infection* or neonatal infection*).mp.	5569
7	1 or 2 or 3 or 4 or 5 or 6	464742
8	(chest indrawing* or chest-indrawing* or chest in-drawing* or chest wall indrawing* or chest wall in-drawing* or chest retraction*).mp.	294
9	((pneumon* adj3 diag*) or (pneumon* adj3 class*) or (pneumon* adj3 symptom*) or (pneumon* adj3 sign*) or respiratory symptom* or respiratory sign* or (illness* adj3 class*) or (sick* adj3 class*) or ("disease*" adj3 class*) or imci class*).mp.	30177
10	8 or 9	30374
11	exp Child/	2041759
12	exp Infant/	1203385
13	exp Infant, Newborn/	644621
14	exp Pediatrics/	61864
15	(child* or infant* or p?ediatric* or neonat* or newborn*).mp.	3215678
16	11 or 12 or 13 or 14 or 15	3216279
17	"Sensitivity and Specificity"/	361762
18	Clinical Competence/	100905
19	(accura* or correct* or valid*).mp.	1814703
20	(sensitiv* or specific* or kappa).mp.	4548342
21	(performance or agreement or skill* or quality or ability or competen*).mp.	3153256
22	(gold standard or gold-standard or reference standard).mp.	77802
23	17 or 18 or 19 or 20 or 21 or 22	7901722
24	7 and 10 and 16 and 23	1611
25	limit 24 to humans	1578
26	limit 25 to yr="1990 -Current"	**1480**
**B. Name of Database: Embase 1980 to 2022 Week 02**		
1	exp pneumonia/	341881
2	exp respiratory tract infection/	428898
3	pneumon*.mp.	465152
4	(respiratory tract infection* or acute respiratory infection* or acute respiratory tract infection* or arti or lower respiratory infection* or lrti).mp.	118922
5	newborn disease/	19406
6	infant disease/	6669
7	childhood disease/	60524

8	(child* illness* or newborn illness* or neonatal illness* or newborn infection* or neonatal infection*).mp.	13587
9	1 or 2 or 3 or 4 or 5 or 6 or 7 or 8	864448
10	(chest indrawing* or chest-indrawing* or chest in-drawing* or chest wall indrawing* or chest wall in-drawing* or chest retraction*).mp.	447
11	((pneumon* adj3 diag*) or (pneumon* adj3 class*) or (pneumon* adj3 symptom*) or (pneumon* adj3 sign*) or respiratory symptom* or respiratory sign* or (illness* adj3 class*) or (sick* adj3 class*) or ("very severe disease*" adj3 class*) or imci class*).mp.	50433
12	10 or 11	50739
13	child/	1795037
14	exp infant/	994749
15	newborn/	522982
16	pediatrics/	79876
17	(child* or infant* or p?ediatric* or neonat* or newborn*).mp.	3603233
18	13 or 14 or 15 or 16 or 17	3607289
19	"sensitivity and specificity"/	417620
20	diagnostic accuracy/	275754
21	accuracy/	171060
22	validation study/	93843
23	clinical competence/	63882
24	(accura* or correct* or valid*).mp.	3170554
25	(sensitiv* or specific* or kappa).mp.	5973386
26	(performance or agreement or skill* or quality or ability or competen*).mp.	5243537
27	(gold standard or gold-standard or reference standard).mp.	159197
28	19 or 20 or 21 or 22 or 23 or 24 or 25 or 26 or 27	11633749
29	9 and 12 and 18 and 28	2950
30	limit 29 to human	2820
31	limit 30 to yr="1990 -Current"	**2772**
**C. Name of database: Web of Science Core Collection (1990-current)**		
1	pneumon* (Topic)	245,030
2	"respiratory tract infection*" OR "acute respiratory infection*" OR "acute respiratory tract infection*" OR arti OR "lower respiratory infection*" OR lrti (Topic)	31,337
3	"child* illness*" OR "newborn illness*" OR "neonatal illness*" OR "newborn infection*" OR "neonatal infection*" (Topic)	5,255
4	#3 OR #2 OR #1	271,244
5	"chest indrawing*" OR "chest-indrawing*" OR "chest in-drawing*" OR "chest wall indrawing*" OR "chest wall in-drawing*" OR "chest retraction*" (Topic)	271
6	(pneumon* NEAR/3 diag*) OR (pneumon* NEAR/3 class*) OR (pneumon* NEAR/3 symptom*) OR (pneumon* NEAR/3 sign*) OR "respiratory symptom*" OR "respiratory sign*" OR (illness* NEAR/3 class*) OR (sick* NEAR/3 class*) OR (“very severe disease*” NEAR/3 class*) OR "imci class*" (Topic)	38,434
7	#6 OR #5	38,602
8	child* OR infant* OR p$ediatric* OR neonat* OR newborn* (Topic)	2,819,346
9	accura* OR correct* OR valid* (Topic)	5,349,940
10	sensitiv* OR specific* OR kappa (Topic)	7,214,629
11	performance OR agreement OR skill* OR quality OR ability OR competen* (Topic)	10,040,939
12	“gold standard” OR gold-standard OR “reference standard” (Topic)	94,627
13	#12 OR #11 OR #10 OR #9	18,562,007
**15**	#4 AND #7 AND #8 AND #13	1,562
**16**	#15 (1990-2022)	**1,561**
**D. Name of database: Scopus**		
1	TITLE-ABS-KEY (pneumon*)	490,551
2	TITLE-ABS-KEY ("respiratory tract infection*" OR "acute respiratory infection*" OR "acute respiratory tract infection*" OR arti OR "lower respiratory infection*" OR lrti)	122,733
3	TITLE-ABS-KEY ("child* illness*" OR "newborn illness*" OR "neonatal illness*" OR "newborn infection*" OR "neonatal infection*")	13,587

4	#1 OR #2 OR #3	592,794
5	TITLE-ABS-KEY ("chest indrawing*" OR "chest-indrawing*" OR "chest in-drawing*" OR "chest wall indrawing*" OR "chest wall in-drawing*" OR "chest retraction*")	375
6	TITLE-ABS-KEY ((pneumon* W/3 diag*) OR (pneumon* W/3 class*) OR (pneumon* W/3 symptom*) OR (pneumon* W/3 sign*) OR "respiratory symptom*" OR "respiratory sign*" OR (illness* W/3 class*) OR (sick* W/3 class*) OR ("very severe disease*" W/3 class*) OR "imci class*")	47,713
7	#5 OR #6	47,959
8	TITLE-ABS-KEY (child* OR infant* OR p?ediatric* OR neonat* OR newborn*)	4,407,745
9	TITLE-ABS-KEY (accura* OR correct* OR valid*)	7,369,662
10	TITLE-ABS-KEY (sensitiv* OR specific* OR kappa)	9,950,237
11	TITLE-ABS-KEY (performance OR agreement OR skill* OR quality OR ability OR competen*)	13,697,403
12	TITLE-ABS-KEY ("gold standard" OR gold-standard OR "reference standard")	169,737
13	#9 OR #10 OR #11 OR #12	25,582,116
14	#4 AND #7 AND #8 AND #13	2,719
15	#14 (1990-2010)	1,022
16	#14 (2011-2022)	1,554
17	#15 OR #16	**2,576**

Table S2. Extraction of data from eligible studies

**A. Identification of the study**		
1	Study ID	
2	Title	
3	First author	
4	Year of publication	
5	Name of the journal	
6	Study Region – Country	
**B. Study methods**		
7	Study Settings – Facility/community	
8	Study period	
9	Sampling method	
**C. Population characteristics**		
10	Age group	
11	Number of children	
12	Number of observations	
13	Inclusion criteria	
14	Exclusion criteria	
**D. Index test**		
15	Index test used	
16	Number of health workers	
17	Education	
18	Training	
19	Blinding	
**E. Reference standard**		
20	Reference standard used	
21	Qualification	
22	Blinding	
**F. Chest indrawing assessment**		
23	Sequence of assessment	
24	Time interval between index test and reference standard	
**G. Study findings**		
25	Prevalence of chest indrawing in the sample	

26	True positive	
27	False positive	
28	False negative	
29	True negative	
30	Sensitivity (95% CI)	
31	Specificity (95% CI)	
32	Positive predictive value (95% CI)	
33	Negative predictive value (95% CI)	
34	Accuracy (95% CI)	
40	Notes	

Table S3. QUADAS-2

DOMAIN 1: PATIENT SELECTION

A. Risk of Bias

- ❖ Was a consecutive or random sample of patients enrolled? Yes/No/Unclear
- ❖ Was a case-control design avoided? Yes/No/Unclear
- ❖ Did the study avoid inappropriate exclusions? Yes/No/Unclear

Could the selection of patients have introduced bias? RISK: LOW/HIGH/UNCLEAR

B. Concerns regarding applicability

Is there concern that the included patients do not match the review question? **CONCERN: LOW/HIGH/UNCLEAR**

DOMAIN 2: INDEX TEST(S)

A. Risk of Bias

- ❖ Were the index test results interpreted without knowledge of the results of the reference standard? Yes/No/Unclear
- ❖ If a threshold was used, was it pre-specified? Yes/No/Unclear

Could the conduct or interpretation of the index test have introduced bias? RISK: LOW/HIGH/UNCLEAR

B. Concerns regarding applicability

Is there concern that the index test, its conduct, or interpretation differ from the review question? **CONCERN: LOW/HIGH/UNCLEAR**

DOMAIN 3: REFERENCE STANDARD

A. Risk of Bias

- ❖ Is the reference standard likely to correctly classify the target condition? Yes/No/Unclear
- ❖ Were the reference standard results interpreted without knowledge of the results of the index test? Yes/No/Unclear

Could the reference standard, its conduct, or its interpretation have introduced bias? RISK: LOW/HIGH/UNCLEAR

B. Concerns regarding applicability

Is there concern that the target condition as defined by the reference standard does not match the review question? **CONCERN: LOW /HIGH/UNCLEAR**

DOMAIN 4: FLOW AND TIMING

A. Risk of Bias

- ❖ Was there an appropriate interval between index test(s) and reference standard? Yes/No/Unclear
- ❖ Did all patients receive a reference standard? Yes/No/Unclear
- ❖ Did patients receive the same reference standard? Yes/No/Unclear
- ❖ Were all patients included in the analysis? Yes/No/Unclear

Could the patient flow have introduced bias? RISK: LOW/HIGH/UNCLEAR

Table S4. Excluded studies with reasons for exclusion

Sl	Author, Year	Title	Reason for exclusion
1	Abayneh, 2020	Improving the Assessment and Classification of Sick Children according to the Integrated Management of Childhood Illness (IMCI) Protocol at Sanja Primary Hospital, Northwest Ethiopia: A Pre-Post Interventional Study	No reference standard
2	Aftab, 2018	Improving community health worker performance through supportive supervision: a randomised controlled implementation trial in Pakistan	Unclear reference standard
3	Bandyopadhyay, 2003	Are Primary Health Workers Skilled Enough to Assess the Severity of Illness Among Young Infants?	No disaggregated data on chest indrawing
4	Biswas, 2011	Skill of Frontline Workers Implementing Integrated Management of Neonatal and Childhood Illness: Experience from a District of West Bengal, India	No disaggregated data on chest indrawing
5	Baynes, 2018	Quality of Sick Child-Care Delivered by Community Health Workers in Tanzania	No data for chest indrawing accuracy
6	Bjornstad, 2014	Determining the quality of IMCI pneumonia care in Malawian children	No disaggregated data on chest indrawing
7	Daka, 2020	Quality of clinical assessment and management of sick children by Health Extension Workers in four regions of Ethiopia: A cross-sectional survey	No data for chest indrawing accuracy
8	Darnstadt, 2009	Validation of community health workers' assessment of neonatal illness in rural Bangladesh	No data for chest indrawing accuracy
9	Elimian, 2020	Comparing the accuracy of lay diagnosis of childhood malaria and pneumonia with that of the revised IMCI guidelines in Nigeria	No disaggregated data on chest indrawing
10	Gadomski, 1993	Assessment of respiratory rate and chest indrawing in children with ARI by primary care physicians in Egypt	Health workers' performance not assessed
11	Getachew, 2019	Health Extension Workers' diagnostic accuracy for common childhood illnesses in four regions of Ethiopia: a cross-sectional study	No disaggregated data on chest indrawing
12	Getachew, 2021	Association between a complex community intervention and quality of health extension workers' performance to correctly classify common childhood illnesses in four regions of Ethiopia	No disaggregated data on chest indrawing
13	Gilroy, 2013	Quality of sick child care delivered by Health Surveillance Assistants in Malawi	No data for chest indrawing accuracy
14	Gove, 1997	Integrated management of childhood illness: field test of the WHO/UNICEF training course in Arusha, United Republic of Tanzania	No disaggregated data on chest indrawing
15	Hadi, 2001	Diagnosis of pneumonia by community health volunteers: experience of BRAC, Bangladesh	No disaggregated data on chest indrawing
16	Hadi., 2003	Management of acute respiratory infections by community health volunteers: experience of Bangladesh Rural Advancement Committee (BRAC)	No disaggregated data on chest indrawing
17	Huicho, 2008	How much does quality of child care vary between health workers with differing durations of training? An observational multicountry study	No data for chest indrawing accuracy
18	Kahigwa, 2002	Inter-observer variation in the assessment of clinical signs in sick Tanzanian children	No reference standard
19	Kallander, 2006	Can community health workers and caretakers recognise pneumonia in children? Experiences from western Uganda	No data for chest indrawing accuracy
20	Kalyango, 2012	Performance of community health workers under integrated community case management of childhood illnesses in eastern Uganda	No data for chest indrawing accuracy

Sl	Author, Year	Title	Reason for exclusion
21	Kolstad, 1997	The integrated management of childhood illness in western Uganda	No data for chest indrawing accuracy
22	Li, 2012	Evaluation of Short Term Integrated Management of Childhood Illness Training on the Clinical Competency of Village Doctors in Yunnan, China	No disaggregated data on chest indrawing
23	Mukanga, 2011	Can lay community health workers be trained to use diagnostics to distinguish and treat malaria and pneumonia in children? Lessons from rural Uganda	No disaggregated data on chest indrawing
24	Nalwadda, 2013	Community health workers – a resource for identification and referral of sick newborns in rural Uganda	Health workers assessed chest indrawing from videotaped cases
25	Narang, 2007	Clinico-epidemiological profile and validation of symptoms and signs of severe illness in young infants (<60 days) reporting to a district hospital	No data for chest indrawing accuracy
26	Onono, 2018	Community case management of lower chest indrawing pneumonia with oral amoxicillin in children in Kenya	No disaggregated data on chest indrawing
27	Sarswati, 2020	'Know-Can' gap: gap between knowledge and skills related to childhood diarrhoea and pneumonia among frontline workers in rural Uttar Pradesh, India	Health workers assessed chest indrawing from videotaped cases
28	Sgewade, 2013	Integrated Management of Neonatal and Childhood Illness (IMNCI): Skill Assessment of Health and Integrated Child Development Scheme (ICDS) Workers to Classify Sick Under-five Children	No data for chest indrawing accuracy
29	Some, 2017	Integrated eDiagnosis approach: Assessing the quality of the management of children illnesses in Burkina Faso	Conference proceeding
30	The Young Infants Clinical Signs Study Group, 2008	Clinical signs that predict severe illness in children under age 2 months: a multicentre study	Health workers' performance not assessed
31	Uwemedimo, 2018	Distribution and determinants of pneumonia diagnosis using Integrated Management of Childhood Illness guidelines: a nationally representative study in Malawi	No disaggregated data on health workers' performance
32	Weber, 1997	Evaluation of an algorithm for the integrated management of childhood illness in an area with seasonal malaria in the Gambia	No disaggregated data on chest indrawing
33	Yalçın, 2018	Agreement Between Integrated Management of Childhood Illness and Final Diagnosis in Acute Respiratory Tract Infections	Health workers' performance not assessed
34	Zeitz et al., 1993	Community Health Worker Competency in Managing Acute Respiratory Infections of Childhood in Bolivia	Health workers assessed chest indrawing from videotaped cases

Table S5. Data used for the meta-analysis

author	year	age	tp	fp	fn	tn
Brady	1993	0-3 months	15	17	24	144
Brewster	1993	1-59 months	11	15	22	175
Mulholland	1992a	2-59 months	13	39	15	241
Mulholland	1992b	2-59 months	8	5	11	267
Perkins	1997	2-59 months	91	41	69	1204
Simoes	1992a	2-59 months	14	6	27	284
Simoes	1992b	2-59 months	26	12	12	254
